# Extrapulmonary and Other *Pneumocystis jirovecii* Infections Diagnosed via Fungal Cell-Free DNA Using Giant Magnetoresistance Detection

**DOI:** 10.3390/jof12060435

**Published:** 2026-06-15

**Authors:** Jo-Anne H. Young, Karam M. Obeid, Minggan Li, Hannah Sweet, Jacob Panten, Tiffany J. Huwe, Xiaoying Liu

**Affiliations:** 1 Department of Medicine, Division of Infectious Disease and International Medicine, University of Minnesota, MMC 250, 420 Delaware St. SE, Minneapolis, MN 55455, USA; 2 Zepto Life Technology, Inc., Saint Paul, MN 55114, USA; minggan.li@zeptolife.com (M.L.); hannah.sweet@zeptolife.com (H.S.); jacob.panten@zeptolife.com (J.P.); tiffany.huwe@zeptolife.com (T.J.H.); xiaoying.liu@zeptolife.com (X.L.)

**Keywords:** cell-free DNA, cfDNA, *Pneumocystis jirovecii*, giant magnetoresistance, GMR

## Abstract

Clinical suspicion and diagnosis of *Pneumocystis* infections rely on a combination of compatible clinical, radiologic, and diagnostic findings. We noted an extrapulmonary *Pneumocystis* infection when evaluating residual plasma samples from patients with possible invasive fungal infections using cell-free DNA (cfDNA) Giant Magnetoresistance (GMR) (FungiFlex^®^, Zepto Life Technology). A man, immunocompromised for 27 years due to kidney transplantations, developed fever and rising aminotransferase enzyme levels during transient escalation of immunosuppressive therapy. Except for an elevated (1,3)-beta-D-glucan, further laboratory testing did not find the highly suspected histoplasmosis. Daily plasma blood samples for cfDNA fungal GMR testing were examined for histoplasmosis; however, the fungal GMR signal was only positive for *Pneumocystis jirovecii* in all four samples. In retrospect, the transient escalation of immunosuppressive dosing created a temporary net state of over-immunosuppression that was permissive for *Pneumocystis*. Additionally, the assay corroborated pulmonary pneumocystosis for a second patient, was negative for a third patient with colonization, and was negative for a fourth patient when fevers correlated with an abdominal bacterial process. Within the exploratory nature of the findings, we find that the reference standard for defining *Pneumocystis* is imperfect and may not accurately identify all cases. cfDNA testing may supplement existing laboratory testing and improve the diagnosis of *Pneumocystis*.

## 1. Introduction

Most patients with *Pneumocystis jirovecii (P. jirovecii)* infection present with pneumonia [1]. The critical point for developing *Pneumocystis* pneumonia typically occurs when the CD4 T-cell count drops to less than 200 cells/mm^3^ [2]. At this point, prophylactic antibiotics such as trimethoprim-sulfamethoxazole (TMP-SMX) are recommended to prevent infection [3].

Clinicians may suspect *Pneumocystis* pneumonia from a constellation of typical symptoms (fever, cough, shortness of breath), chest imaging findings, and laboratory markers in an immunocompromised host. Non-specific serum biomarkers such as lactate dehydrogenase (reflecting lung injury) and (1,3)-beta-D-glucan (BDG, a component of fungal cell walls) are suggestive of infection [4,5,6]. Still, a *Pneumocystis* pneumonia diagnosis requires specific detection of the fungus using specialized cytologic staining, direct fluorescent antibody staining, or DNA testing. Specimens appropriate for staining include expectorated sputum, induced sputum, or bronchoalveolar lavage fluid. These can also be tested using molecular methods such as polymerase chain reaction (PCR) assays, which are often more sensitive than non-molecular methods [7,8]. A drawback is that PCR assays using specimens from the pulmonary tree also detect colonization [9]. Molecular testing has been used to detect *P. jirovecii* cell-free DNA (cfDNA) in patient plasma [10,11,12].

Zepto Life Technology has developed a novel multiplex molecular diagnostic platform that uses Giant Magnetoresistance (GMR) for detection of PCR-amplified cfDNA from multiple invasive fungal species [13,14]. Our center has been providing residual blood samples from patients with possible invasive fungal infections to evaluate Zepto’s FungiFlex^®^ system, which leverages GMR for direct detection and identification of invasive fungal pathogens directly from plasma. The panel of evaluable fungi includes *P. jirovecii* and 21 other fungi. The patients presented in this case series show that the assay agreed with one case of pulmonary *Pneumocystis*, was negative for a patient with colonization, and was negative when another patient’s fevers correlated with an abdominal bacterial process. We were surprised to discover a case of extrapulmonary *Pneumocystis* infection when the suspected infection was histoplasmosis.

This article addresses the diagnostic challenges associated with invasive fungal infections in immunocompromised patients. We explore the emerging application of plasma fungal cfDNA testing in several clinically complex scenarios and present informative contrasting cases that highlight the potential diagnostic difficulties in distinguishing colonization from active infection.

## 2. Materials and Methods

Approval for the collection of residual body fluid specimens for GMR testing was obtained by the University of Minnesota Institutional Review Board under study protocol #00020540. GMR testing is conducted in Zepto Life Technology’s (Zepto, St Paul, MN, USA) Clinical Laboratory Improvement Amendments (CLIA) certified microbiology laboratory. Pre-PCR operations are conducted in a HEPA-filtered cleanroom to prevent environmental contamination. In this study, we use GMR for the detection of PCR amplicons from trace amounts of fungal cfDNA. Samples are collected from patients who have actual or suspected infections from one of 22 fungal organisms on the GMR assay panel (*Aspergillus flavus*, *A. fumigatus*, *A. niger*, *A. terreus*, *Fusarium verticillioides/oxysporum*, *F. solani*, *Mucor circinelloides*, *Rhizopus oryzae*, *R. microsporus*, *Rhizomucor miehei*, *Cunninghamella bertholletiae*, *Scedosporium* spp./*Lomentospora prolificans*, *Candida albicans*, *C. auris*, *Nakaseomyces glabratus* [*C. glabrata*], *Pichia kudriavzevii* [*C. krusei*], *C. tropicalis*, *C. parapsilosis*, *Histoplasma capsulatum*, *Cryptococcus neoformans*, *Coccidioides immitis/posadasii*, and *P. jirovecii*). At the time of this study, a mold panel including the first 11 targets listed above was validated as a laboratory-developed test; the remaining targets, including *P. jirovecii* and *C. parapsilosis*, were for research use only.

Residual samples of venous whole blood in 3 mL K2EDTA vacutainer tubes (Greiner Bio-One round-bottom polystyrene Vacuette^®^ tubes, Monroe, NC, USA) from patients with possible fungal infection were saved after each morning’s complete blood count (CBC). Sample tubes were stored upright for 1–4 days at refrigerator temperature (4 °C). Transport to the Zepto Life Technology laboratory occurred within 1 h in a cooler. After arrival, samples were centrifuged at 1500× *g* for 10 min to separate plasma from whole blood. The plasma was then aliquoted into sterile 2 mL tubes and stored at −80 °C until sample testing.

cfDNA was extracted from each plasma sample using the Maxwell^®^ CSC Rapid ccfDNA Kit (AS1580, Promega, Madison, WI, USA) with the automated Maxwell^®^ CSC Instrument (AS6000, Promega, Madison, WI, USA). Plasma from a healthy human donor was used as a negative control in each extraction run.

The purified cfDNA was screened for invasive fungal pathogens using Zepto’s FungiFlex^®^ assay as previously described [13]. In summary, the purified cfDNA is subdivided into a set of multiplexed PCRs. During PCR, species-specific fungal sequences (ITS, 18S, or 5S) are amplified and biotinylated. Additionally, each multiplexed PCR amplifies a human sequence as an internal control. The resulting PCR products are then combined and selectively digested using an exonuclease to produce single-stranded biotinylated amplicons. The resulting single-stranded biotinylated DNA sample is then loaded into the proprietary Zepto detection cartridge. The proprietary Zepto reader drives a microfluidic process to flow the sample over a 36-plex GMR sensor, which is surface-coated with oligonucleotide probes specific to target fungal species. As the sample flows over the sensor, the signal-stranded amplicons selectively hybridize with probes. After hybridization, magnetic beads are introduced, which bind selectively to the amplicon–probe complexes. Sensors with bound magnetic beads generate signals, measured in relative magnetic resistance units (RMUs), with magnitude corresponding to the variable levels of hybridized complexes on the GMR sensor surface. The locations of signals generated across the sensor determine the species identification. Positivity thresholds are in the 200 to 600 RMU range for each fungal target (200 RMUs for *P. jirovecii*, 300 RMUs for *C. parapsilosis*).

## 3. Results

### 3.1. Kidney Transplant Recipient with Extrapulmonary Pneumocystis

Our patient is a 64-year-old man with a history of IgA nephropathy requiring kidney transplantation in 1997. He had an intermediate-intensity induction immunosuppression protocol for a second kidney transplant from a living donor in 2024, with maintenance immunosuppression consisting of tacrolimus, mycophenolate mofetil, and prednisone. Two severe infections after the second transplant, polyomavirus BK (BK virus) viremia and V1 trigeminal herpes zoster ophthalmicus, led to serial decreases in mycophenolate mofetil dosing. At the first anniversary of the second kidney transplant, his CD4 count was 731 cells/mm^3^, and TMP-SMX prophylaxis for *Pneumocystis* pneumonia was discontinued.

He had ongoing squamous cell carcinomas treated with wide local skin excision as well as radiation therapy. Due to the recurrent skin cancers, everolimus was substituted for tacrolimus with a 4-day overlap before stopping tacrolimus. An everolimus level on Day+4 of therapy was above the therapeutic goal, so the dose was reduced. On Day+11 of therapy, a second everolimus level was even higher, so the dose was further reduced. He developed a fever of 104 °F thirteen days after starting everolimus, for which he was seen in the emergency department on Day+15 and Day+17. Upon admission to the hospital on Day+17, everolimus was held. Fevers continued, inflammatory markers were high, and empiric antibacterial therapy with cefepime was not effective. Chest radiographs on Day+15 and Day+17 demonstrated no acute airspace disease.

**Figure 1:**
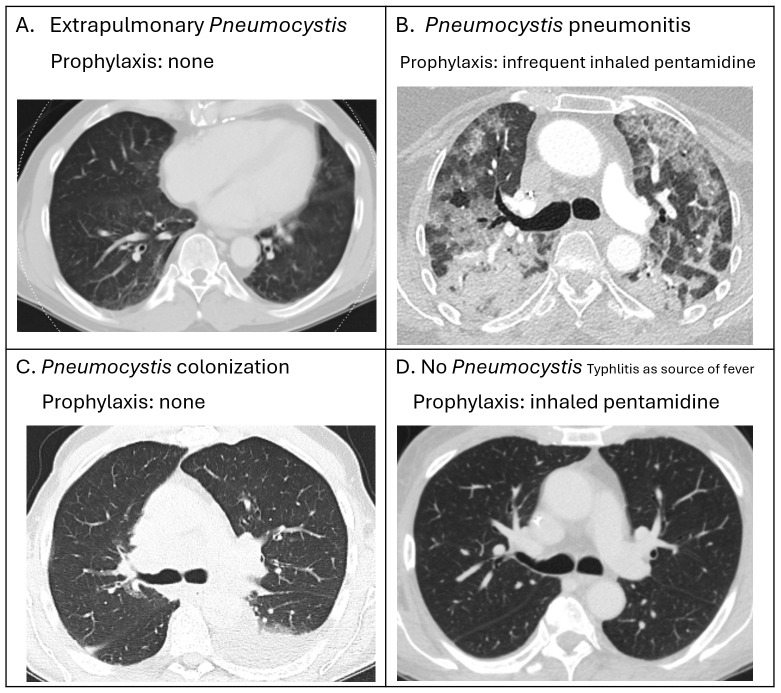
CT imaging findings for the four case patients. The abnormal CT scan in pneumonitis (**B**) is caused by massive alveolar inflammation and filling, whereas colonization (**C**) represents a low-burden presence of the fungus without significant inflammatory response. The finding of relatively normal lung CT scans in patients with colonization (**B**), extrapulmonary infection (**A**), or no *Pneumocystis* infection (**D**) is in stark contrast to the severe imaging abnormalities in pneumonitis (**B**), and is a perfect illustration of the difference between the presence of the organism and active, tissue-damaging infection.

With inpatient bloodwork showing elevation of (1,3)-beta-D-glucan, there was strong clinical suspicion for histoplasmosis, as he was an active outdoorsman in the Mississippi River Valley region of the United States with community-onset fever and rising liver aminotransferase enzymes. Liver ultrasound on Day+17 demonstrated normal parenchyma with a smooth contour. Computed tomography imaging of the abdomen and pelvis on Day+19 showed a normal appearance of the liver, biliary system, and lymph nodes. Views of the lower lungs showed non-acute fibroatelectatic subpleural changes (Figure 1A).

After further fungal testing was sent but had not yet resulted, he had an immediate response in fever to the addition of itraconazole therapy with a loading dose, although this coincided with holding immunosuppression. Inflammatory markers improved by the third day of itraconazole therapy, and he was discharged from the hospital using lower doses of immunosuppression. Liver enzymes were normal within one month. *Histoplasma* (sent to Associated Regional and University Pathologists Inc, Salt Lake City, UT, USA) and *Blastomyces* (sent to MiraVista Diagnostics, Indianapolis, IN, USA) urine antigens later resulted in negative results (Table 1). He completed three months of itraconazole therapy.

Retrospective plasma samples and subsequent GMR testing showed that four daily plasma samples from Day+17 through to Day+20 were negative for *Histoplasma*. Unexpectedly, the GMR signal was positive for *P. jirovecii* in each sample tested (Table 1). The GMR assay detects polymicrobial fungal infections, so the lack of finding *Histoplasma* was indeed felt to reflect its absence, since *Pneumocystis* and *Histoplasma* co-infections can occur [15]. Other fungal targets were also negative.

In retrospect, tacrolimus levels were higher than desired, and the everolimus dosing did not need to be as high as it was started; the extra-pulmonary *Pneumocystis* infection was self-limited by lowering immunosuppression. He did not receive *Pneumocystis*-directed therapy, and no *Pneumocystis* prophylaxis was started at clinic follow-up visits. CD4 count was not checked.

### 3.2. Liver Transplant Recipient with Pulmonary Pneumocystis and Candidemia

Our patient is a 75-year-old woman with a history of hypertension, type 2 diabetes, and liver transplantation in 2010 for hepatitis C and hepatocellular carcinoma. She developed post-transplant lymphoproliferative disorder in 2023, requiring chemotherapy, and a port-a-cath was in place for treatments. She was using inhaled pentamidine infrequently as *Pneumocystis* prophylaxis, with the last two doses being given 24 and 101 days before the *Pneumocystis* diagnostic test.

She was admitted to the intensive care unit with a cough, progressive hypoxic respiratory failure requiring intubation, and diffuse bilateral infiltrates. CT imaging showed multifocal bilateral airspace opacities, more consolidated in the lower lobes and more ground-glass in the bilateral upper lobes (Figure 1B). Day−1 BDG later returned >500 pg/mL, consistent with fungal infection, including *Pneumocystis* infection, so micafungin was started on Day+0. She was diagnosed with breakthrough *Pneumocystis* pneumonia infection based on *P. jirovecii* PCRs of bronchoscopy fluid on Day+0, and the procedure was complicated by pneumothorax requiring chest tube placement twice. Lactate dehydrogenase was elevated to 619 U/L on Day+2, 602 on Day+26, and 438 on Day+28. She was treated with TMP-SMX, high-dose steroids, and interventions for septic shock. She received 22 days of *Pneumocystis* therapy (TMP-SMX was followed by clindamycin and primaquine), followed by single-strength TMP-SMX for secondary prophylaxis. Fevers responded to her cares, and micafungin was discontinued on Day+8. Retrospective plasma samples and subsequent GMR testing showed positive GMR signals for *P. jirovecii* on Days+8, +11, and +13, with six daily negative tests thereafter (Table 2).

Fevers recurred by Day+18, with blood cultures negative on Day+18 and Day+25. Urine cultures on Day+26 and Day+27 grew *C. parapsilosis* complex, and blood culture on Day+31 recovered *C. parapsilosis* complex. Interestingly, the retrospective plasma samples that underwent GMR testing with the FungiFlex^®^ assay repeatedly showed the presence of a second fungal organism in blood. Elevated GMR signals for *C. parapsilosis* complex were found for six of nine samples tested; two near the threshold for a positive test and four well within the positive range (GMR ≥ 300) (Table 2). These samples pre-dated the known candiduria and candidemia by up to 3 weeks. She had risk factors for candidal infection with indwelling lines and antibiotic use during her prolonged hospital course in the intensive care unit. The antifungal agent micafungin was used for 8 days due to the positive BDG assay.

After a subarachnoid hemorrhage, she transitioned to comfort care. She died 32 days after the *P. jirovecii* PCR test. Upon review of her case, the retrospective FungiFlex^®^ result correlated with the clinical picture, helping to further support patient management and adding clarity to the diagnostic pathway.

### 3.3. Lymphoma Patient Colonized with Pneumocystis

Our patient is a 75-year-old man treated for lymphoma in 2013. As a physically active outpatient, he developed a dry cough and wheezing, followed by night sweats, in 2025. CT imaging and lung biopsies showed relapse of lymphoma. In addition to lymphoma changes, the lung parenchyma showed subtle changes in infection: mild diffuse bronchial wall thickening, ground-glass opacity, and consolidation in the right apex, patchy bronchovascular ground-glass opacities in the right upper lobe, and anterior right middle lobe ground-glass opacities (Figure 1C). Bronchoscopy to rule out infection (Day+0) before starting chemotherapy cultured *Moraxella catarrhalis*, for which he was treated with ceftriaxone. Fungal culture resulted in *Trichoderma* species; without symptoms of fever or consolidative pneumonia, it was interpreted as an environmental saprobe. Cytologic examination of the bronchoscopy lavage fluid showed lymphoma cells; fungal staining was not performed.

He was admitted to the hospital on Day+4 to start chemotherapy. The lavage fluid from Day+0 resulted in *P. jirovecii* by PCR, followed by negative BDG testing using the BDG assay on Day+5 and Day+8. He started *Pneumocystis* prophylaxis with TMP-SMX 1 single strength daily and fungal prophylaxis with posaconazole on Day+5 for one month, since he was initiating chemotherapy. He was discharged from the hospital on Day+6. Three retrospective plasma samples from Day+4, Day+5, and Day+6 showed negative fungal GMR testing signals (Table 3).

### 3.4. Leukemia Patient with Neutropenic Fever and Eventual Typhlitis, but No Pneumocystis

**Figure 2:**
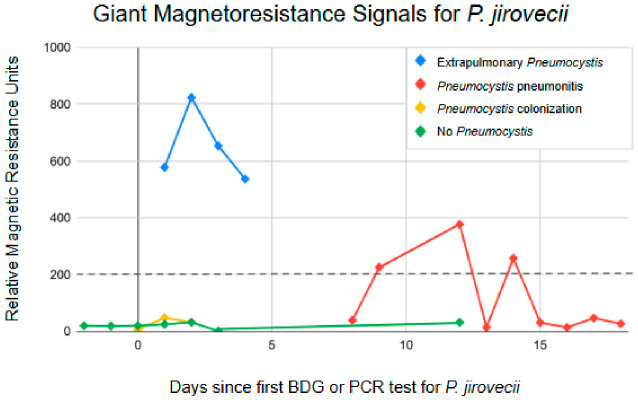
*P. jirovecii* GMR signals for the four case patients. Zero on the x-axis marks the day of the first BDG or PCR test for *P. jirovecii* conducted during each clinical episode. The dotted line at 200 RMUs marks the positivity threshold for *P. jirovecii* signals.

Our patient is a 72-year-old man who had a 2-year-long battle with acute lymphoblastic leukemia treatments, including a reduced-intensity allogeneic transplant in 2024, followed by CD19 Chimeric antigen receptor T-cell therapy with obecabtagene autoleucel. He was cytopenic, dependent on colony-stimulating factor and transfusion support. Sequential efforts at *Pneumocystis* prophylaxis with TMP-SMX and dapsone were difficult for him, so he was taking monthly inhaled pentamidine for over 15 months. He was noted to have a fever during outpatient clinic visits. BDG testing using the Fungitell assay was negative (Table 4). Minimal elevation of lactate dehydrogenase (LDH) of 322 U/L (range, 0–250) on Day+0 was felt to represent his leukemia. He trialed atovaquone as a fourth medication option for *Pneumocystis* with no response to the fever. CT imaging found diffuse mild edematous colonic wall thickening, greatest along the cecum/ascending colon, suspicious for typhlitis, so antibacterial agents were continued. The lungs had minimal changes related to a potential infection—some multifocal ground-glass opacities, most prominently in the right upper lobe and left lower lobe (Figure 1D). His fever responded while taking antibacterial agents to address proctocolitis, with concurrent improvement in his white blood cell count out of the neutropenic range. GMR testing of six retrospective plasma samples showed negative signals for all fungi tested, including *Pneumocystis* (Table 4, Figure 2).

## 4. Discussion

This case series highlights that plasma cfDNA can be used to verify *Pneumocystis* pneumonia, contribute to a diagnosis of early-stage extrapulmonary *Pneumocystis* (EPP), help to differentiate colonization from infection (no test alone can distinguish between colonization and infection), and remain negative when there is no *Pneumocystis* infection. The GMR sensor provides desirable sensitivity and specificity for detecting *P. jirovecii* from minimally invasive plasma specimens, rather than from invasive and contamination-prone sampling of the pulmonary tree.

We were particularly intrigued to discover a case of EPP infection when the suspected infection was histoplasmosis. Finding an unexpected fungus when suspecting another is highly valuable because it enables rapid, targeted therapy for potentially life-threatening infections, prevents the misuse of ineffective treatments, and aids in distinguishing infection from colonization. Multiplex diagnostic tests are critically important, offering fast detection of a suspected fungal pathogen and even simultaneous detection of multiple pathogens, as was our case of a liver transplant recipient with the finding of candidemia in addition to *Pneumocystis*.

EPP without concurrent *Pneumocystis* pneumonia is a rare but critical condition. Due to the rarity of EPP, a high index of clinical suspicion is a primary driver of diagnosis, especially in the context of unexplained systemic illness in immunocompromised patients. The current gold standard testing for EPP relies on identifying the organism through histopathology or microscopy (Gomori Methenamine Silver stain) of tissue samples, which is often delayed and invasive. It is considered a significant disadvantage in clinical diagnosis when clinicians excessively delay or hesitate over whether to perform a tissue biopsy, as it can delay critical treatment initiation and negatively affect patient outcomes. Applying *Pneumocystis* testing to body fluid sources from the pulmonary tree is an imperfect situation for finding EPP when there is no pulmonary involvement [16].

While biopsy procedures carry risks like infection or bleeding, they are often considered the “gold standard” for establishing a definitive diagnosis and determining the precise attributes of a disease. Since the best available test for finding *Pneumocystis* in extrapulmonary sites is histopathology [17,18,19,20], a new sensitive and specific laboratory test for EPP without *Pneumocystis* pneumonia would ideally identify disease at earlier stages than with the previous gold standard. EPP diagnosis may require a composite standard, combining multiple imperfect tests into a robust standard. This would be better than waiting for the disease to fully manifest or for treatment response to indicate a suspected diagnosis. Serum BDG is not an acceptable gold standard; while it is a sensitive, non-invasive biomarker that can be elevated in EPP, it lacks specificity for *Pneumocystis* alone (it indicates pan-fungal infection). Emerging approaches combine PCR, BDG, and molecular resistance testing to identify “probable” cases earlier than waiting for tissue pathology samples, particularly for inoperable or high-risk patients.

EPP often has a lower organism burden than pneumonia, making highly sensitive methods like PCR and metagenomic next-generation sequencing (mNGS) essential [21]. CfDNA PCR testing has shown promising sensitivity (100%, 10/10) and specificity (93%, 127/136) for detecting *P. jirovecii* infections [10]. While molecular tests offer speed, histopathology may remain crucial for some cases to visualize the tissue-level impact of the infection.

The risk timeframe for *Pneumocystis* infections varies based on the form of immunosuppression, so prophylaxis is offered using a protocolized approach [22,23,24]. Following solid organ transplantation, immunosuppression levels are highest in the first year to prevent organ rejection. Lung transplant recipients continue lifelong *Pneumocystis* prophylaxis, while other organ recipients may have prophylaxis discontinued once immunosuppression is lowered to maintenance levels [25]. Guidelines from the American Society of Transplantation recommend a standard duration of 6 to 12 months for *Pneumocystis* prophylaxis among kidney transplant recipients, as was the case with our kidney transplant patient [26]. Lymphopenia is associated with late-onset *P. jirovecii* pneumonia in solid organ transplantation [27]. Our liver transplant recipient had a breakthrough infection while receiving inhaled pentamidine, a known risk of this route of delivering prophylaxis, although she had not been compliant with obtaining these treatments every month.

Prophylaxis was not restarted [22,28,29] for our long-term kidney transplant recipient who had overlapping use of immunosuppressive agents, since his last CD4 count was over 700. Additionally, the timeframe for tacrolimus and everolimus overlap would be short, on the order of two weeks. However, high everolimus blood levels created a temporary net-state of over-immunosuppression that was permissive for *Pneumocystis*. As levels of immunosuppressive medications dropped to their intended levels, by holding the medication and then restarting at a much lower dose, his own immune system was able to control the extrapulmonary *Pneumocystis* infection without direct therapy. Since chest imaging studies did not show pulmonary infection and he did not have pulmonary symptoms, we believe his diagnosis should be classified as an early-stage EPP infection. This infection responded to lowering of immunosuppression without the use of targeted *Pneumocystis* therapy.

The literature on EPP has been actively growing since the 2000s, with many contemporary studies and case reports published in the last 5–10 years showing increased recognition of EPP in non-HIV immunocompromised patients (transplant, cancer, autoimmune) and the impact of new diagnostic tools like PCR [15,18,19]. DNA analysis has reclassified the human pathogen as *P. jirovecii*, distinguishing it from animal pathogens. Use of topical/localized prophylactic medications such as aerosolized pentamidine might contribute to the relative increase in extrapulmonary disease. While reports identified EPP (then *P. carinii*) [30] in the 1980s, modern research focuses on its increasing prevalence outside HIV, genetic insights, and varied sites like lymph nodes, spleen, and bone marrow, with significant work from the last few years detailing new occurrences and management.

The remaining three cases collectively illustrate the clinical utility of multiplex cfDNA invasive fungal testing in immunocompromised patients with complex infectious presentations. In case 2, notably, the FungiFlex^®^ GMR assay retrospectively detected both *P. jirovecii* and *C. parapsilosis* in plasma rescue samples in a liver transplant recipient, with the *Candida* signal preceding clinical recognition of candidemia by up to three weeks, a significant interval, although we do not wish to overstate the clinical implications given the retrospective nature of this analysis. This finding underscores a well-recognized diagnostic gap: conventional blood cultures detect only approximately 50% of invasive candidiasis cases and have a median time to positivity of 2–3 days, even when positive, whereas molecular assays may identify circulating fungal DNA earlier in the disease course. The premature discontinuation of micafungin on Day+8, driven by clinical improvement attributed solely to (*P. jirovecii* pneumonia) PJP treatment, highlights how co-infections can be masked in critically ill immunocompromised patients and how multiplex molecular detection of multiple invasive fungal organisms might have a potentially meaningful impact on management.

Case 3 provides an interesting example of deciphering invasive fungal disease vs. colonization for the patient with a newly diagnosed relapse of lymphoma. The positive *Pneumocystis* PCR from bronchoalveolar lavage, in the setting of negative BDG testing, absence of typical radiographic findings of PJP (the ground-glass opacities), and negative cfDNA signal in plasma, collectively support colonization over active infection. We would like to clearly acknowledge that plasma cfDNA negativity alone cannot exclude active disease, and that diagnostic interpretation relies on integrating clinical presentation, imaging, BDG testing, PCR findings, and therapeutic response. The decision to initiate TMP-SMX prophylaxis dosing rather than therapeutic dosing was clinically appropriate, as colonization in the setting of impending chemotherapy-induced immunosuppression carries a risk of progression to active disease.

Case 4 serves as a true negative control, demonstrating the specificity of both BDG and GMR testing. In this profoundly immunosuppressed patient with neutropenic fever ultimately attributed to typhlitis, all six retrospective plasma GMR samples were negative for all fungal targets, including *Pneumocystis*, and BDG was also negative. The mild LDH elevation was appropriately attributed to underlying leukemia rather than PJP.

There are hypothetical clinical benefits if real-time assay results had been available. We speculate that, taken together, these cases demonstrate that the FungiFlex^®^ GMR assay may have offered clinically meaningful advantages over conventional diagnostics in distinguishing active *Pneumocystis* infection (case 3.b, with positive plasma signals) from colonization (case 3.c, with negative plasma signals) and non-fungal etiologies (case 3.d), while simultaneously detecting co-pathogens such as *Candida* species that might otherwise go unrecognized until later in the clinical course.

## 5. Limitations

There are limitations to FungiFlex^®^ GMR testing while it is still part of a research platform. Because GMR testing was conducted after the clinical episodes and results were not available in real-time, it was not possible to run confirmatory diagnostic testing for the kidney transplant recipient with the unexpected *Pneumocystis* diagnosis. The plasma samples used for the four case patients were rescued retrospective convenience samples obtained from leftover complete blood count (CBC) test K2EDTA tubes. Due to the retrospective study design, we could not always access samples taken during the onset of symptoms or on the same day as standard-of-care testing, which would have allowed stronger diagnostic comparisons.

In general, pre-analytical variability accounts for the majority of diagnostic testing errors. False positives could result from environmental contamination. The opening of the tubes for CBC testing increased the risk of introducing environmental contaminants, although over the course of testing for this study, we did not observe *P. jirovecii* or other fungal contamination. For the two patients where GMR testing identified *P. jirovecii*, pathogen cfDNA was found in multiple samples (4/4 and 3/9), increasing our confidence in the findings.

False negatives could result from transient cfDNAemia, specimen degradation, or low input volume. The stability of cfDNA in whole blood collected without preservatives (e.g., standard EDTA tubes) is generally observed at 2 to 8 h at room temperature [31,32] and up to 24 to 48 h when refrigerated [33,34,35] before lysis and cell death occurs, which some samples in the series may have been subject to. Prompt separation of plasma is often required for optimal assay performance. Temperature extremes, delays in processing, or storage issues were not noted and human internal controls were detected in each sample. Remnant plasma volumes in this study varied from 0.5 to 1.5 mL. The FungiFlex^®^ mold panel, for example, was validated using 2 mL plasma. Inputting less plasma reduces the likelihood of pathogen cfDNA detection; however, in the case of EPP, *P. jirovecii* was detected in each sample. Despite these limitations, the FungiFlex^®^ assay provided clinical results that supported the diagnostic interpretation for patients presented herein.

## 6. Conclusions

This exploratory and hypothesis-generating look-back investigation finds that cfDNA can be used to point towards *Pneumocystis* pneumonia and early-stage EPP. It may assist in differentiating colonization from infection, and remain negative when there is no *Pneumocystis* infection. In particular, we would like to emphasize the diagnosis of early-stage EPP when there is an imperfect gold standard for EPP. With the development of sensitive molecular diagnostic testing assays, extrapulmonary *Pneumocystis* infection may come to be recognized with increasing frequency.

The clinical course of our kidney transplant recipient provides insight into the natural history of EPP. The kidney transplant case remains speculative, since no tissue diagnosis, extrapulmonary specimen confirmation, or definitive microbiological evidence were presented, and the patient improved following multiple simultaneous interventions, including reduction in immunosuppression and empirical antifungal therapy. We interpret this as a possible or suspected occult extrapulmonary *Pneumocystis* infection detected by plasma cfDNA.

## Figures and Tables

**Table 1:** Giant magnetoresistance testing results for the kidney transplant recipient with extrapulmonary *Pneumocystis,* in relation to case-patient attributes. Days refer to days since the start of everolimus immunosuppression.

Day	Everolimus Dose	Everolimus Level, Goal 4–6	Fever	Other	GMR Result for *P. jirovecii* (Positivity Threshold 200)
Day+0	1.25 mg BID				
Day+4	1.25 mg BID	7.3 ng/mL		Tacrolimus overlap period completes	
Day+6	↓ to 1 mg BID				
Day+11	1 mg BID	10.0 ng/mL	Outpatient fevers begin		
Day+13	↓ to 0.75 BID		Outpatient fevers		
Day+14	0.75 BID		Emergency room visit for fever		
Day+15	0.75 BID		Outpatient fevers	Absolute lymphocyte count 600 cells/mm^3^	
Day+16	Hospital admission				
	Held	6.7 ng/mL	>101 °F	CRP 251	
				BDG > 500	
Day+17	Held	8.2 ng/mL	>100 °F	ESR > 140	578
Day+18	Held; itraconazole added		>101 °F	*Histoplasma* and *Blastomyces* testing negative	824
Day+19	0.25 BID	3.9 ng/mL	No fever		654
Day+20	0.25 BID		No fever	CRP 114 ESR 105	537
	Hospital discharge				
Day+23	0.25 BID	10.1 ng/mL			
Day+28	↓ to 0.25 daily				
Day+32	↓ to 0.25 every other day				
Day+39	0.25 every other day	4.8 ng/mL		Itraconazole 1.8 + 2.1 = 3.9	
				BDG 289	
Day+87	0.25 every other day			ESR 31	
Day+102	0.25 every other day	5.8 ng/mL		Itraconazole 3.5 + 3.9 = 7.4	
Day+142				BDG 54	

Notes: GMR = giant magnetoresistance. BID = twice daily. Down arrow (↓) = dose decrease. °F = Fahrenheit. CRP = C-reactive protein, mg/L (range, <5). BDG = (1,3)-beta-D-glucan assay, normal range < 31 pg/mL. ESR = erythrocyte sedimentation rate, mm/h (range, 0–20). Itraconazole level is a combined value of the drug level and the active metabolite. The goal itraconazole trough concentration for treating histoplasmosis is a level greater than or equal to 1.0 mcg/mL (or mg/L), as measured by high-performance liquid chromatography. Blue background color = important information correlates related to *Pneumocystis* infection. Tan background color = offset highlight related to hospital admission or discharge.

**Table 2:** Giant magnetoresistance testing results for the liver transplant patient undergoing chemotherapy for post-transplant lymphoma, who developed pulmonary *Pneumocystis* and *C. parapsilosis* infection. Day refers to the date from which the *Pneumocystis* pneumonia diagnostic test was taken.

Day	Temperature	Diagnostic Testing	GMR Result for *P. jirovecii* (Positivity Threshold 200)	GMR Result for *C. parapsilosis* (Positivity Threshold 300)
Day−101	Inhaled pentamidine treatment administered			
Day−24	Inhaled pentamidine treatment administered			
Day−2	Hospital admission for acute hypoxic respiratory failure			
Day−1	102.7 °F; piperacillin-tazobactam start	Blood and urine culture negative BDG > 500		
Day+0	102.4 °F; micafungin start	Blood and BAL culture negative BAL PCR + for *Pneumocystis*; chest tube		
Day+1	98.2 °F	Trimethoprim-sulfamethoxazole start for *Pneumocystis* treatment		
Day+2	98.6 °F	Second chest tube insertion for pneumothorax that developed after Day+0 bronchoscopy		
Day+3	98.4 °F	Piperacillin-tazobactam complete		
Day+7	101.5 °F; levofloxacin start	Blood culture negative	39	254
Day+8	100.4 °F; micafungin stop		226	20
Day+11	100.4 °F		377	833
Day+12	98.4 °F		14	4967
Day+13	98.1 °F		258	810
Day+14	99.3 °F		31	29
Day+15	99 °F		14	1376
Day+16	99.3 °F		47	−8
Day+17	97.9 °F		27	250
Day+18	100.5 °F; levofloxacin stop	Blood culture negative		
Day+25	100.2 °F	Blood culture negative		
Day+26	99.6 °F	Urine culture with *C. parapsilosis* complex		
Day+27	99.7 °F	Urine culture with *C. parapsilosis* complex		
Day+31	100.4 °F	Blood culture with *C. parapsilosis* complex		
Day+32	Patient passes away			

Notes: GMR = giant magnetoresistance. °F = Fahrenheit. BDG = (1,3)-beta-D-glucan assay, normal range < 31 pg/mL. BAL = bronchoalveolar lavage. Blue background color = important information correlates related to *Pneumocystis* infection. Orange background colors = important information correlates related to *Candida* infection. Tan background color = offset highlight related to hospital admission or discharge.

**Table 3:** Giant magnetoresistance testing results for the lymphoma patient starting chemotherapy, who had colonization of the pulmonary tree with *Pneumocystis* and *Trichoderma*. Day refers to the date from which the *Pneumocystis* pneumonia diagnostic test was taken.

Day	Culture	Other	GMR Result for *P. jirovecii* (Positivity Threshold 200)	LDH (Reflects Lymphoma)
Day−3				771
Day+0	BAL bacterial culture with *Moraxella* BAL fungal culture *Trichoderma*	BAL PCR + for *Pneumocystis*		
Day+1				978
Day+4	Hospital admission to start chemotherapy			
		BDG < 31	6	
Day+5			48	411
	Ceftriaxone started to treat *Moraxella*			
	Posaconazole started for secondary fungal prophylaxis			
	Trimethoprim-sulfamethoxazole started for secondary *Pneumocystis* prophylaxis			
Day+6			32	
	Hospital discharge			
Day+8		BDG < 31		
Day+11				244
Day+35	Posaconazole and trimethoprim-sulfamethoxazole prescriptions complete			

Notes: GMR = giant magnetoresistance. LDH = lactate dehydrogenase, in U/L, with a normal range of 0–250. BAL = bronchoalveolar lavage. PCR = polymerase chain reaction. BDG = (1,3)-beta-D-glucan assay, normal range < 31 pg/mL. Blue background color = important information correlates related to *Pneumocystis* infection. Tan background color = offset highlight related to hospital admission or discharge.

**Table 4:** Giant magnetoresistance testing results for the leukemia patient with neutropenic fever and eventual typhlitis, but no *Pneumocystis*. Day refers to the date from which the (1,3)-beta-D-glucan assay diagnostic test was taken.

Day	Fever	Culture	Other	GMR Result for *P. jirovecii* (Positivity Threshold 200)	White Blood Cell Count
Day−2	Hospital admission for neutropenic fever, antibacterial agents, and micafungin start				
	102.9 °F	Blood negative	CRP 44	20	0.47
Day−1	100.9 °F			19	0.32
Day+0	102.5 °F		BDG < 31 Atovaquone start	20	0.26
Day+1	102.2 °F	Blood negative	CRP 90	25	0.16
Day+2	100.6 °F		CRP 85	32	0.17
		CT scan shows typhlitis: diffuse, mild edematous wall thickening of the colon, greatest along the cecum/ascending colon Minimal changes in the lung parenchyma of ground-glass opacities with mixed consolidation, most prominently in the right upper lobe and left lower lobe			
Day+3	99.6 °F		Histoplasma antigen negative	1	0.22
Day+4	98.2 °F				0.28
Day+5	97.7 °F				0.49
Day+6	97.8 °F				0.53
Day+7	Isavuconazole replaces micafungin		CRP 24		0.78
Day+8	98.3 °F				1.06
Day+9	98.2 °F		CRP 16		1.10
Day+10	98.9 °F				1.13
Day+12	98.7 °F			32	1.40
	Hospital discharge with levofloxacin, atovaquone, and isavuconazole				

Notes: GMR = giant magnetoresistance. °F = Fahrenheit. CRP = C-reactive protein, mg/L (range, <5). BDG = (1,3)-beta-D-glucan assay, normal range < 31 pg/mL. White blood count normal range 4.0–11.0 × 10^3^/uL. Blue background color = important information correlates related to *Pneumocystis* infection. Tan background color = offset highlight related to hospital admission or discharge.

## Data Availability

The original contributions presented in the study are included in the article; further inquiries can be directed to the corresponding author.
